# The Surgical Apgar Score can help predict postoperative complications in femoral neck fracture patients: a 6-year retrospective cohort study

**DOI:** 10.1186/s40981-018-0205-y

**Published:** 2018-09-10

**Authors:** Atsushi Kotera

**Affiliations:** Department of Anesthesiology, Kumamoto Central Hospital, 955 Muro, Ozu-machi Kikuchi-gun, Kumamoto, 869-1235 Japan

**Keywords:** Femoral neck surgery, Surgical Apgar Score, Postoperative mortality

## Abstract

**Introduction:**

The postoperative mortality rate following a femoral neck fracture remains high. The Surgical Apgar Score (SAS), based on intraoperative blood loss, the lowest mean arterial pressure, and the lowest heart rate, was created to predict 30-day postoperative major complications. Here, we evaluated the relationship between the SAS and postoperative complications in patients who underwent femoral neck surgeries.

**Methods:**

We retrospectively collected data from patients with femoral neck surgeries performed in 2012–2017 at Kumamoto Central Hospital. The variables required for the SAS and the factors presumably associated with postoperative complications including the patients’ characteristics were collected from the medical charts. Intergroup differences were assessed with the *χ*^2^ test with Yates’ correlation for continuity in category variables. The Mann-Whitney *U* test was used to test for differences in continuous variables. We assessed the power of the SAS value to distinguish patients who died ≤ 90 days post-surgery from those who did not, by calculating the area under the receiver operating characteristic curve (AUC).

**Results:**

We retrospectively examined the cases of 506 patients (94 men, 412 women) aged 87 ± 6 (range 70–102) years old. The 90-day mortality rate was 3.4% (*n* = 17 non-survivors). There were significant differences between the non-survivors and survivors in body mass index (BMI), the presence of moderate to severe valvular heart disease, albumin concentration, the American Society of Anesthesiologists (ASA) classification, and the SAS. The 90-day mortality rate in the SAS ≤ 6 group (*n* = 97) was 10.3%, which was significantly higher than that in the SAS ≥ 7 group (*n* = 409), 1.7%. The AUC value to predict the 90-day mortality was 0.70 for ASA ≥ 3 only, 0.71 for SAS ≤ 6 only, 0.81 for SAS ≤ 6 combined with ASA ≥ 3, and 0.85 for SAS ≤ 6 combined with albumin concentration < 3.5 g/dl, BMI ≤ 20, and the presence of moderate to severe valvular heart disease.

**Conclusions:**

Our results suggest that the SAS is useful to evaluate postoperative complications in patients who have undergone a femoral neck surgery. The ability to predict postoperative complications will be improved when the SAS is used in combination with the patient’s preoperative physical status.

## Background

The number of patients with femoral neck fractures is increasing worldwide due to the aging of many populations [1]. Femoral neck fractures can introduce various serious physical complications such as pneumonia subsequent to prolonged immobility. Thus, femoral neck fractures can be an important cause of postoperative mortality, and the reported mortality rates of patients with femoral neck fractures at 1 month, 6 months, and 1 year are 2.7, 10.8, and 16.3%, respectively [2]. A better understanding of postoperative complications in patients with femoral neck surgeries is therefore needed. The risk factors related to postoperative mortality after femoral neck surgeries are generally thought to be advanced age, dementia, low level of daily life activity, male gender, anemia, low value body mass index (BMI), and underlying cardiac-related diseases [3, 4]. Most of these factors reflect a patient’s preoperative status and do not include the patient’s intraoperative status. The Surgical Apgar Score (SAS), which is based on the patient’s intraoperative blood loss, lowest mean arterial pressure, and lowest heart rate, has been reported to be useful for predicting postoperative complications in general and in vascular surgeries [5]. In the present study, we retrospectively analyzed the relationship between the SAS and postoperative complications in the patients who underwent femoral neck surgeries over a 6-year period at our hospital, and our findings revealed the usefulness of the SAS for predicting postoperative complications in such patients.

## Methods

### Patients

Approval for this retrospective study (no. 2017-013) was provided by the Ethical Committee of Kumamoto Central Hospital, Kumamoto, Japan on December 27, 2017. Patients who underwent osteosynthesis or hip hemiarthroplasty for a femoral neck fracture under general or spinal anesthesia performed between January 1, 2012, and December 31, 2017, at Kumamoto Central Hospital were eligible for this study.

### Protocol design

This was a single-center, retrospective cohort study. All data were pre-existing data obtained from the patients’ medical records and did not include any personal information that would identify any of the patients. Informed consent from the patients was therefore waived, based on the Ethical Guidelines for Epidemiological Studies issued jointly by the Ministry of Health, Labour and Welfare and the Ministry of Education, Culture, Sports, Science, and Technology of Japan.

### Data collection

Data were collected based on the variables required for the SAS (Table 1). The SAS is calculated at the end of an operation and is the sum of the points from each category. The calculated total SAS using the three variables ranges from 0 to 10 points, and the SAS value increases as the patient’s outcome improves at 30 days post-surgery. The data of the patient’s intraoperative blood loss, lowest mean arterial pressure, and lowest heart rate (which are required for the calculation of the SAS) were obtained from the intraoperative anesthesia records.

**Table 1:** Variables required for the Surgical Apgar Score

	Number of points				
	0	1	2	3	4
Blood loss (g)	> 1000	601–1000	101–600	≤ 100	–
Lowest mean arterial pressure (mmHg)	< 40	40–54	55–69	≥ 70	–
Lowest heart rate (beats per min)	> 85	76–85	66–75	56–65	≤55

The Surgical Apgar Score is the sum of the points from each category

At our hospital, the determination of the value of a patient’s intraoperative blood loss is based on both the weight of gauze used and the volume of the suction used. For all patients, blood pressure was measured noninvasively every 2–5 min and his or her heart rate was monitored continuously throughout the surgical procedure. Factors presumably associated with postoperative complications including patient characteristics were also extracted from the medical charts: patient age, gender, BMI, underlying comorbidities, the results of biochemical examinations of blood (hemoglobin, C-reactive protein, albumin concentration, sodium concentration, and blood urea nitrogen/creatinine ratio), the American Society of Anesthesiologists (ASA) classification, the ejection fraction measured by echocardiography, the presence or absence of moderate to severe valvular heart disease, the partial pressure of oxygen in arterial blood, the presence or absence of dementia, the waiting period prior to surgery, the presence or absence of preoperative fever, the method of anesthesia, the precise type of surgery, and the operation time. At our hospital, the method of anesthesia is left to the individual anesthesiologist. When the patient’s preoperative condition was stable, general anesthesia was the first choice. Conversely, when the patient had one or more serious underlying comorbidities such as chronic heart failure or pulmonary disease, spinal anesthesia was administered.

In this study, we set the 90-day mortality as the primary endpoint and the 30-day postoperative complications as the secondary endpoint. Postoperative complications were included only if the patient had received a medical or interventional treatment. The postoperative complications were recorded in each patient’s medical record. We analyzed the following major postoperative complications described by Gawande et al. [5]: acute renal failure, acute heart failure, bleeding requiring ≥ 4 units of red cell transfusion within 72 h after the operation, cardiac arrest requiring cardiopulmonary resuscitation, deep venous thrombus, myocardial infarction, pneumonia, stroke, surgical site infection, and sepsis. We did not include superficial surgical site infections or urinary tract infections as major postoperative complications.

We divided the patients into two groups: those who were still alive at 90 days post-surgery (the survivor group) and those who had not survived as of 90 days post-surgery (the non-survivor group), and we analyzed the patients’ characteristics between the two groups. We also divided the patients into the low-risk group (with SAS values≥ 7) and high-risk group (SAS ≤ 6) using a previously established threshold [6], and we compared the 90-day mortality and 30-day postoperative complications between these two groups.

### Statistical analysis

All statistical analyses were carried out using the software program Excel Tokei 2012 (Social Survey Research Information, Tokyo). Intergroup differences were assessed with the *χ*^2^ test with Yates’ correlation for continuity in category variables. The Mann-Whitney *U* test was used to test for differences in continuous variables. Differences of *p* < 0.05 were considered significant. Descriptive data are presented as the mean ± standard deviation (range).

We assessed the power of a model to distinguish patients who died within 90 days after the femoral neck fracture surgery from those who did not by calculating the area under the receiver operating characteristic curve (AUC) [7]. Concerning category variables, we used the sum of the points from each category as explanatory variables to calculate the AUC. In our analysis, the presence of each category indicates 1 point and the absence indicates 0 points. When there are five categories in a prediction model, the sum points range from 0 to 5 points. Concerning continuous variables, we used the value itself as explanatory variable to calculate the AUC. The AUC value ranged from 0.5 to 1.0, and the greater the AUC, the better the model. An AUC of 1.0 indicates a perfect model that has 100% sensitivity and 100% specificity. An AUC of 0.5 indicates a model that is completely ineffective in differentiating between real cases and non-cases.

## Results and discussion

We retrospectively analyzed the cases of 506 patients (94 men [18.6%] and 412 women [81.4%]) operated for a femoral neck fracture during the study period. The demographic data of the patients are summarized in Table 2. Their age was 87 ± 6 (range 70–102) years old. The most common comorbidity was hypertension. The incidence of the presence of moderate to severe valvular heart disease was 18.0% (91 of the 506 patients). The frequency of general anesthesia was performed in 74.3% (376 of the 506 patients). The SAS value was 7.5 ± 1.1 (range 4–10), and each variable was as follows: the blood loss was 48 ± 96 g (range 10–860 g), the lowest mean arterial pressure (MAP) was 57 ± 10 mmHg (range 37–112 mmHg), and the lowest heart rate was 59 ± 10 beats per min (bpm) (range 38–101 bpm).

**Table 2:** Demographic data of the patients and comparison of clinical characteristics between the non-survivors and survivors

	All the patients (*N* = 506)	Non-survivors (*N* = 17)	Survivors (*N* = 489)	*p* value
Age (year)	87 ± 6 (70–102)	88 ± 7 (74–99)	87 ± 6 (70–102)	0.403
Male	94 (18.6)	4 (23.5)	90 (18.4)	0.593
Body mass index	20.4 ± 3.2 (13.7–32.0)	18.9 ± 4.1 (14.2–30.8)	20.5 ± 3.2 (13.7–32.0)	0.018
Comorbidities				
Hypertension	283 (55.9)	9 (52.9)	274 (56.0)	0.801
Old cerebral infarction	63 (12.5)	4 (23.5)	59 (12.1)	0.159
Chronic heart failure	61 (12.1)	2 (11.8)	59 (12.1)	0.970
Diabetes mellitus	58 (9.6)	3 (17.6)	55 (11.2)	0.416
Chronic renal failure	21 (4.2)	1 (5.9)	20 (4.1)	0.716
Chronic obstructive pulmonary disease	20 (4.0)	2 (11.8)	18 (3.7)	0.093
Rheumatoid arthritis	18 (3.6)	2 (11.8)	16 (3.3)	0.063
Old myocardial infarction	15 (3.0)	1 (5.9)	14 (2.9)	0.471
Insertion of cardiac pacemaker	9 (2.9)	0 (0)	9 (1.8)	0.573
Hemoglobin (g/dl)	11.1 ± 1.8 (6.1–16.8)	10.4 ± 2.2 (6.6–15.6)	11.2 ± 1.8 (6.1–16.8)	0.114
C-reactive protein (mg/dl)	2.6 ± 3.5 (0.0–24.3)	2.7 ± 3.3 (0.0–12.5)	2.6 ± 3.5 (0.0–24.3)	0.653
Albumin concentration (g/dl)	3.5 ± 0.5 (2.1–4.6)	3.2 ± 0.5 (2.2–4.0)	3.5 ± 0.5 (2.1–4.6)	0.006
Sodium concentration (mEq/L)	139 ± 4 (120–162)	140 ± 5 (131–147)	139 ± 4 (120–162)	0.391
Blood urea nitrogen/creatinine ratio	26.7 ± 9.1 (7.8–83.2)	29.0 ± 12.4 (10.4–56.3)	26.7 ± 8.9 (7.8–83.2)	0.601
ASA classification ≥ 3	249 (49.2%)	15 (88.2%)	234 (47.8%)	0.001
Ejection fraction (%)	64 ± 9 (39–89)	63 ± 10 (45–80)	64 ± 9 (39–89)	0.441
Moderate to severe valvular heart disease	91 (18.0)	7 (41.2)	84 (17.2)	0.011
PaO_2_ (mmHg)	76 ± 11 (45–104)	78 ± 10 (60–97)	76 ± 12 (45–104)	0.365
Dementia	244 (48.2)	10 (58.8)	234 (47.8)	0.374
Waiting period for surgery(day)	9.0 ± 3.3 (2–20)	9.5 ± 4.0(5–18)	9.0 ± 3.3(2–20)	0.922
Preoperative fever (≥ 38°)	151 (29.8%)	8 (47.1%)	143 (29.2%)	0.110
General anesthesia	376 (74.3)	11 (64.7)	365 (74.6)	0.357
Spinal anesthesia	130 (25.7)	6 (35.3)	124 (25.4)	
Hip hemiarthroplasty	101 (20)	5 (29.4)	96 (19.6)	0.321
Osteosynthesis	405 (80)	12 (70.6)	393 (80.4)	
Operation time (min)	47 ± 25 (13–190)	47 ± 24 (18–104)	47 ± 25 (13–190)	0.952

The data are given as patient’s number (%) or the mean ± standard deviation (range)

*PaO*_*2*_ partial pressure of oxygen in arterial blood gas analysis

The 90-day mortality rate was 3.4% (17 of the 506 patients). The results of our comparison of clinical characteristics between the 17 non-survivors and the 489 survivors are also shown in Table 2. There were significant differences between the two groups in BMI, the presence of moderate to severe valvular heart disease, the albumin concentration, and the ASA classification. The BMI in the non-survivors was 18.9 ± 4.1 (range 14.2–30.8), which was significantly lower than that in the survivors at 20.5 ± 3.2 (range 13.7–32.0). The incidence of moderate to severe valvular heart disease in the non-survivors was 41.1% (7 of the 17 patients), which was significantly higher than that in the survivors at 17.2% (84 of the 489 patients). The albumin concentration in the non-survivors was 3.2 ± 0.5 g/dl (range 2.2–4.0 g/dl), which was significantly lower than that in the survivors at 3.5 ± 0.5 g/dl (range 2.1–4.6 g/dl). The incidence of the ASA classification ≥ 3 in the non-survivors was 88.2% (15 of the 17 patients), which was significantly higher than that in the survivors at 47.8% (234 of the 489 patients). The incidences of old cerebral infarction, chronic obstructive pulmonary disease (COPD), and rheumatoid arthritis tended to be more frequent in the non-survivors, but the differences were insignificant.

The results of our comparison of the SAS values between the 17 non-survivors and the 489 survivors are shown in Table 3. The SAS in the non-survivors was 6.4 ± 1.1 (range 4–8), which was significantly lower than that in the survivors at 7.5 ± 1.1 (range 4–10). Among the three variables of the SAS, the lowest MAP in the non-survivors was 54 ± 17 mmHg (range 39–97 mmHg), which was significantly lower than that in the survivors at 57 ± 10 mmHg (range 37–113 mmHg).

**Table 3:** Comparison of the Surgical Apgar Score between non-survivors and survivors

	All the patients (*N* = 506)	Non-survivors (*N* = 17)	Survivors (*N* = 489)	*p* value
Surgical Apgar score	7.5 ± 1.1 (4–10)	6.4 ± 1.1 (4–8)	7.5 ± 1.1 (4–10)	< 0.001
Blood loss (g)	48 ± 96 (10–860)	54 ± 66 (10–195)	48 ± 97 (10–860)	0.439
Lowest mean arterial pressure (mmHg)	57 ± 10 (37–112)	54 ± 17 (39–97)	57 ± 10 (37–113)	0.010
Lowest heart rate (beats per min)	59 ± 10 (38–101)	64 ± 13 (48–90)	59 ± 10 (38–101)	0.112

The 30-day postoperative complications are listed in Table 4. The rate of postoperative complications was 21.7% (110 of the 506 patients), and pneumonia was the most common postoperative complication (41 of the 506 patients). The causes of 90-day mortality were as follows: eight patients died of pneumonia, six patients died of postoperative heart failure, and three patients died of sepsis.

**Table 4:** Proportion of postoperative major complications

Postoperative major complications	*N* (% of the 30-day postoperative complications)	*N* (% of the 90-day mortality)
Pneumonia	41 (8.1)	8 (1.6)
Venous thrombus	26 (5.1)	
Surgical site infection	10 (2.0)	
Postoperative heart failure	9 (1.8)	6 (1.2)
Sepsis	5 (1.0)	3 (0.6)
Bleeding requiring ≥ 4 units of red cell transfusion	4 (0.8)	
Stroke	2 (0.4)	
Acute myocardial infarction	2 (0.4)	
others	11 (2.2)	
Total	110 (21.7)	17 (3.4)

The data are given as patient’s number (%)

The results of our comparison of clinical characteristics between the 97 patients in the SAS ≤ 6 group (the high-risk group) and the 409 patients in the SAS ≥ 7 group (low-risk group) are shown in Table 5. There were significant differences between the two groups in the incidence of underlying COPD and the operation time. The frequency of COPD in the SAS ≤ 6 group was 9.3% (9 of the 97 patients), which was significantly higher than that in the SAS ≥ 7 group at 2.7% (11 of the 409 patients). The operation time in the SAS ≤ 6 group was 53 ± 30 min (range 16–190 min), which was significantly longer than that in the SAS ≥ 7 group at 46 ± 24 min (range 13–167 min).

**Table 5:** Comparison of clinical characteristics between the SAS value ≤ 6 and SAS value ≥ 7 patients

	SAS value ≤ 6 (*N* = 97)	SAS value ≥ 7 (*N* = 409)	*p* value
Age (year)	87 ± 6 (71–102)	87 ± 6 (70–100)	0.480
Male	14 (14.4)	80 (19.6)	0.243
Body mass index	20.2 ± 3.3 (14.2–30.8)	20.5 ± 3.2 (13.7–32.0)	0.596
Comorbidities			
Hypertension	50 (51.5)	233 (57.0)	0.334
Old cerebral infarction	16 (16.5)	47 (11.5)	0.180
Chronic heart failure	9 (9.3)	52 (12.7)	0.350
Diabetes mellitus	7 (7.2)	51 (12.5)	0.144
Chronic renal failure	1 (1.0)	20 (4.9)	0.087
Chronic obstructive pulmonary disease	9 (9.3)	11 (2.7)	< 0.001
Rheumatoid arthritis	2 (2.1)	16 (3.9)	0.376
Old myocardial infarction	3 (3.1)	12 (2.9)	0.934
Insertion of cardiac pacemaker	2 (2.1)	7 (1.7)	0.814
Hemoglobin (g/dl)	11.2 ± 1.9 (6.6–15.6)	11.1 ± 1.8 (6.1–16.8)	0.380
C-reactive protein (mg/dl)	2.8 ± 4.1 (0.0–24.3)	2.6 ± 3.4 (0.0–22.2)	0.740
Albumin concentration (g/dl)	3.6 ± 0.5 (2.1–4.5)	3.5 ± 0.5 (2.3–4.6)	0.316
Sodium concentration (mEq/L)	139 ± 4 (120–162)	139 ± 4 (125–147)	0.790
Blood urea nitrogen/creatinine ratio	26.1 ± 6.7 (13.3–52.9)	26.9 ± 9.5 (7.8–83.2)	0.937
ASA classification ≥ 3	48 (49.4%)	201 (49.1%)	0.952
Ejection Fraction (%)	65 ± 9 (46–89)	64 ± 9 (39–88)	0.529
Moderate to severe valvular disease	15 (15.5)	76 (18.6)	0.472
PaO_2_ (mmHg)	77 ± 11 (51–98)	75 ± 12 (45–104)	0.275
Dementia	47 (48.4)	197 (48.2)	0.959
Waiting period for surgery(day)	9.1 ± 3.6(2–19)	9.0 ± 3.3(2–20)	0.744
Preoperative fever (≥ 38°)	23 (23.7)	128 (31.3)	0.142
General anesthesia	74 (76.3)	302 (73.8)	0.620
Spinal anesthesia	23 (23.7)	107 (26.2)	
Hip hemiarthroplasty	24 (24.7)	77 (18.8)	0.190
Osteosynthesis	73 (75.3)	332 (81.6)	
Operation time (min)	53 ± 30 (16–190)	46 ± 24 (13–167)	0.031

The data are given as patient’s number (%) or the mean ± standard deviation (range)

*PaO*_*2*_ partial pressure of oxygen in arterial blood

**Fig. 1:**
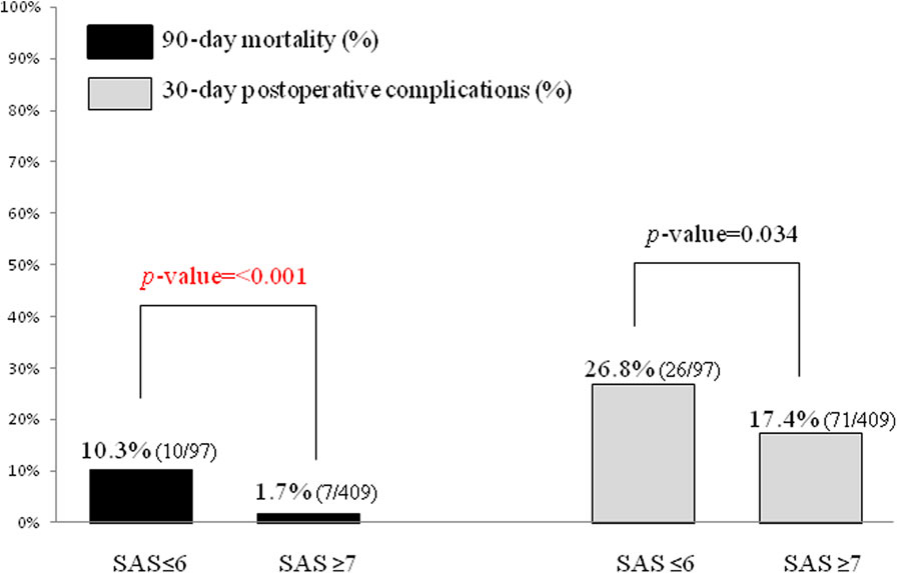
The relationship between the SAS value and the 90-day mortality and 30-day postoperative complications. Patients were divided into two groups: the low-risk group (SAS ≥ 7) and high-risk group (SAS ≤ 6). *Black bar*: The 90-day mortality in each group. *Gray bar*: 30-day postoperative complications

Figure 1 illustrates the relationships between the SAS values and the 30-day postoperative complications and the 90-day mortality. The 90-day mortality rate in the SAS ≤ 6 group (*n* = 97) was 10.3% (10 of the 97 patients), which was significantly higher than that in the SAS ≥ 7 group (*n* = 409) at 1.7% (7 of the 409 patients). The rate of postoperative complications at 30 days in the SAS ≤ 6 group was 26.8% (26 of the 97 patients), which was also significantly higher than that in the patients with SAS ≥ 7 at 17.4% (71 of the 409 patients).

Table 6 shows the AUC to predict the 90-day mortality for each model. For the category variables, the AUC values were 0.70 for ASA ≥ 3 only, 0.77 for significant and marginally significant risk factors in which the *p* value was < 0.1 (i.e., albumin concentration < 3.5 g/dl, BMI ≤ 20, the presence of moderate to severe valvular heart disease, the presence of COPD, and the presence of rheumatoid arthritis), 0.78 for significant risk factors in which the *p* value was < 0.05 (i.e., albumin concentration < 3.5 g/dl, BMI ≤ 20, and the presence of moderate to severe valvular heart disease), 0.71 for SAS ≤ 6 only, 0.81 for SAS ≤ 6 in combination with ASA ≥ 3, 0.84 for SAS ≤ 6 in combination with the significant and marginally significant risk factors, and 0.85 for SAS ≤ 6 in combination with the significant risk factors. For the continuous variables, the AUC values were 0.70 for ASA classification and 0.76 for SAS value.

**Table 6:** Area under the receiver operating characteristic curve to predict the 90-day mortality for each model

Category variables	AUC
Preoperative factor	
ASA classification ≥ 3	0.70
The significant and marginally significant risk factors (*p* value < 0.1)	0.77
Low body mass index (≤ 20)	
Low albumin concentration (< 3.5 g/dl)	
Moderate to severe valvular heart disease	
Chronic obstructive pulmonary disease	
Rheumatoid arthritis	
The significant risk factors (*p* value < 0.05)	0.78
Low body mass index (≤ 20)	
Low albumin concentration (< 3.5 g/dl)	
Moderate to severe valvular heart disease	
Intraoperative factor	
Lowest MAP < 55 mmHg	0.65
Lowest MAP < 55 mmHg and lowest heart rate ≥ 60 beats per min	0.68
SAS value ≤ 6	0.71
Combination of preoperative and intraoperative factor	
SAS value ≤ 6 combined with ASA classification ≥ 3	0.81
SAS value ≤ 6 combined with the significant and marginally significant risk factors	0.84
SAS value ≤ 6 combined with the significant risk factors	0.85
Continuous variables	AUC
ASA classification	0.70
SAS value	0.76

*AUC* area under the receiver operating characteristic curve, *MAP* mean arterial pressure, *SAS* Surgical Apgar Score

Our present analysis revealed that at our hospital, the mortality rates of patients who underwent surgery during the study period for a femoral neck fracture at 30, 90, and 180 days were 0.2, 3.4, and 5.1%, respectively. The rate of postoperative complications in those patients was still high despite the recent advances in surgical and anesthetic techniques; thus, the assessment of postoperative complications in such patients has become increasingly important for both surgeons and anesthesiologists.

Our present retrospective findings indicate that a low BMI, a low albumin concentration, the presence of moderate to severe valvular heart disease, a high ASA classification, and a low SAS value are risk factors for 90-day mortality following a femoral neck fracture. A low BMI and a low albumin concentration indicate malnutrition, and in another femoral neck surgery patient series, malnutrition was reported to be associated with higher rates of postoperative complications, postoperative mortality, and readmissions [8]. Malnutrition is related to delayed bone healing, surgical site infection, and worse functional recovery. Thus, BMI values < 22 were reported to be associated with an increase of almost seven times the mortality at 1 year compared to BMI values > 25 [9]. However, the cutoff values of BMI to diagnose malnutrition differ among studies; for example, ≤ 18.5 reported by Maffulli et al. [10], < 20 reported by Fabian et al. [11], and < 22 reported by Schaller et al. [9].

In the present study, we set the cutoff BMI value as ≤ 20 because the mean value of BMI of all of our patients was almost 20. Although the cutoff value of BMI for malnutrition remains controversial, we propose that a patient’s malnutrition is critically important as it is not only a risk factor for femoral neck fracture but also reduces the patient’s ability to recover his or her pre-fracture functional capacity [12]. However, clinicians should also keep in mind that a high BMI due to an increased fat mass and a decreased muscle mass may often mask the presence of sarcopenia. On the other hand, it is reported that hypoalbuminemia (albumin concentration < 3.5 g/dl) was a risk factor for mortality following femoral neck surgery [13]. That author noted that patients with hypoalbuminemia had higher rates of death, sepsis, and unplanned intubation compared to the patients with normal albumin concentrations. Therefore, in order to predict postoperative complications, it is important to assess the patient’s nutrition status using not only the BMI but also albumin concentration.

The SAS was derived from a retrospective analysis of 303 adult patients who underwent colectomies, and it was validated in two prospective cohorts: 102 colectomy patients and 767 patients who underwent general or vascular surgery [4]. The score is based on only three intraoperative variables, and the calculation of the SAS is simple. The calculated total score using three variables ranges from 0 to 10: the score increases as the outcome improves at the end of 30 days after the surgical procedure. Several research groups noted that the SAS could accurately predict postoperative complications in surgical subspecialties, i.e., neurosurgery [14], esophagectomy [15], and prostatectomy [16]. In the orthopedic field, there are several reports on the relationship between the SAS and postoperative complications, but in the reports, authors stated that the SAS could not accurately predict postoperative complications in patients with hip or knee arthroplasty, lower limb arthroplasty, or femoral neck surgery [6, 17]. Urrutia et al. [18] reported that the SAS had a weak predictive value for 30-day morbidity and mortality in patients who had undergone general orthopedic surgery (except for supine surgery). Regarding the reason why the SAS was not useful for orthopedic patients, it was suggested that the patient’s preoperative physical status might be a stronger predictor than the SAS [6, 17].

However, our present findings demonstrate that the SAS can be useful to assess postoperative complications in the orthopedic field. The discrepancy between our study’s results and those of the abovementioned reports merits discussion. The intraoperative MAP is an important factor for ensuring organ perfusion such as cerebral perfusion, cardiac perfusion, or renal perfusion. An intraoperative MAP value of < 50 mmHg for ≥ 5 min, which reflects a low SAS value, was reported to be correlated with increased 30-day postoperative mortality [19]. Brady et al. [20] also reported that 30-day mortality was significantly associated with > 20 min of MAP < 55 mmHg.

In the non-survivors of the present study, the lowest MAP value of the non-survivors was significantly lower and the incidence of MAP < 55 mmHg was more frequent compared to the values of the survivor group. The difference in the lowest MAP between the non-survivors and survivors was small (just 3 mmHg), and many anesthesiologists feel that the difference is not so serious in a clinical situation. However, Walsh et al. [21] noted that even short durations of intraoperative MAP < 55 mmHg was associated with acute ischemia-reperfusion injury in various organs such as the kidney and heart. Therefore, an intraoperative MAP < 55 mmHg is considered to be one of the risk factors related to postoperative complications.

We also observed a frequent presence of moderate to severe valvular heart disease. It is thus possible that heart failure or decreased organ perfusion associated with intraoperative low mean arterial blood pressure might have caused critical postoperative complications in the patients with moderate to severe valvular heart disease. Therefore, especially in geriatric patients undergoing orthopedic surgery, a low SAS could be closely related to postoperative complications. Anesthesiologists should also keep in mind that the careful management of intraoperative blood pressure may lead to improved postoperative patients’ outcomes. It has been reported that the SAS was not correlated with the postoperative outcomes in orthopedic surgeries performed under regional anesthesia [6, 17, 22]. Nair et al. [22] noted that the initial hypotension that occurred after the administration of a spinal anesthetic was associated with the poor SAS correlation with postoperative complications. In our present study, about three-quarters of the patients were managed with general anesthesia. It was thus possible that a low SAS could be closely related to postoperative complications. We also observed the frequent presence of COPD in our SAS ≤ 6 group. In a femoral neck surgery case, COPD is considered to be a risk factor of postoperative mortality [23]; therefore, we cannot deny the possibility that underlying COPD was associated with the high postoperative mortality and morbidity rate in our low SAS group.

Of course, an assessment based on both a patient’s preoperative physical status and his or her SAS is important. It was reported that the AUC value of a receiver operating characteristic curve was 0.81 for the SAS only and that this value rose to 0.89 when the SAS and the ASA classification was combined [24]. In our study, the AUC value to predict the 90-day mortality was 0.71 for the SAS only, and this value rose to 0.81 when the SAS was combined with ASA ≥ 3. It is notable that in aged patients with femoral neck fractures, an ASA ≥ 3 was reported to be associated with an approx. four to sevenfold increase in postoperative complications compared to ASA ≤ 2 [25]. Nair et al. [22] also noted that the SAS could predict either alone, or in combination with other risk factors, the occurrence of postoperative complications. Therefore, the combination of the SAS and the patient’s preoperative physical status may be an ideal model to predict postoperative complications.

Our study has several limitations. First, the sample size of patients was relatively small, and the data reflect the clinical outcomes at a single center. Our findings may thus not be generalizable for orthopedic patients as a whole. Further studies with larger numbers of patients at multiple centers are needed to test our findings. Second, this study was a retrospective investigation, and prospective studies could be informative. Third, we set the score of each relevant risk factor as 1 point to calculate the AUC for category variables, was done in Apfel’s simplified scoring system [26]. When the score of each relevant risk factor is 1 point, counting the number of risk factors is simple; however, the disadvantage of this system is that the likelihood of the postoperative mortality cannot be derived directly from the number of risk factors. The contribution of each relevant risk factor to the mortality may differ, and thus, we speculate that it would be ideal to process the scores in a logistic model so that the theoretical risks can be calculated. Fourth, the SAS was originally used as a model with the power to predict 30-day postoperative complications, but we used the SAS to predict 90-day mortality instead of 30-day mortality. The reason for this is that the 30-day morality rate of the present patient series at our hospital was 0.2% (1 of the 506 patients), which was so low that a statistical analysis was difficult. However, it was reported that a SAS value ≤ 6 could predict both inpatient postoperative complications and late post-discharge postoperative complications in general surgeries [27]. Brady et al. [20] also noted that intraoperative hypotension might be associated with poor patient outcomes even up to 1 year after surgery. It is thus possible that the SAS could be used to predict not only early but also late postoperative complications. However, we should determine the endpoint of cohort studies carefully.

## Conclusions

The results of our analyses suggest that the SAS is useful to predict postoperative complications in patients who undergo femoral neck surgery. The ability to predict postoperative complications will be improved by combining the SAS with measures of the patient’s preoperative physical status. The calculation of the SAS is simple, and the variables of the SAS are derived from routine intraoperative data available at any hospital. We suggest that the SAS is an easy-to-use model for anesthesiologists, and anesthesiologists should keep in mind that it is important to prevent low mean arterial pressure in order to improve postoperative outcomes.

## Abbreviations

ASA: American Society of Anesthesiologists
AUC: Area under the receiver operating characteristic curve
BMI: Body mass index
COPD: Chronic obstructive pulmonary disease
MAP: Mean arterial pressure
SAS: Surgical Apgar Score
